# Role of TLR4 in the induction of inflammatory changes in adipocytes and macrophages

**DOI:** 10.1080/21623945.2020.1760674

**Published:** 2020-05-13

**Authors:** K. McKernan, M. Varghese, R. Patel, K. Singer

**Affiliations:** Department of Pediatrics and Communicable Disease, University of Michigan Medical School, Ann Arbor, MI, USA

**Keywords:** Toll-like receptors, inflammation, adipose tissue macrophages, adipogenesis, ear mesenchymal stem cells, bone marrow derived dendritic cells, lipopolysaccharide, saturated fatty acids, cytokines

## Abstract

In obesity, high levels of saturated fatty acids (SFAs) contribute to adipose tissue inflammation and dysfunction. Obesity-induced macrophage infiltration leads to insulin resistance, but the adipocyte itself may play a role in generating the inflammatory milieu. Given our recent findings of the role of TLR4 in myeloid biasing in obesity, we next investigated the role of TLR4 in adipocyte generated inflammatory responses to SFAs and lipopolysaccharides. We used WT and *Tlr4^−/-^* ear mesenchymal stem cell derived adipocytes (EMSC Ad) and bone marrow dendritic cells (BMDCs) to evaluate cell specific responses. Our work demonstrates a role for TLR4 in adipocyte- immune cell crosstalk and that SFA derived metabolites from adipocytes may induce proinflammatory stimulation of immune cells in a TLR4 independent manner.

## Introduction

Obesity is associated with a wide variety of comorbidities, including type II diabetes, cardiovascular disease (CVD), and cancer [1–6]. Obese adipose tissue is a site and source for inflammation [7–10] that triggers tissue dysfunction such as impaired adipogenesis [11–13] and excess free fatty acid (FFA) release [14–17]. Adipose dysfunction is closely linked to the recruitment and activation of inflammatory leukocytes [18–21], especially in visceral adipose tissue. In obesity, there is a profound increase in activated CD11 c^+^ pro-inflammatory adipose tissue macrophages (ATMs) within the white adipose tissue (WAT) [22]. ATMs are a prominent source of inflammatory cytokines, such as IL1β, IL6 and tumour necrosis factor α (TNFα) [21,23,24] and chemokines such as MCP1 [25,26] that are important contributors to insulin resistance and overall metabolic syndrome in obesity [7,27].

Among the toll-like receptor (TLR) family members, TLR4 has been recognized as particularly important in terms of adipose tissue inflammation [21,28]. Cytokine profiles in obesity have been observed to be similar to lipopolysaccharide (LPS)-induced TLR4 pathways. TLR4 has also been demonstrated to be involved in promoting alternative macrophage activation, with TLR4 deficient animals on a high fat diet (HFD) displaying a lower incidence of inflammatory CD11 c^+^ macrophages [29]. Previous studies have also indicated a role for TLR4 in the development of adipose tissue fibrosis and insulin resistance in obesity [30,31]. Although TLR4 is typically thought of as a leukocyte receptor, it is also expressed on many non-immune cells, including adipocytes, hepatocytes, and muscle cells [32–34]. Thus, TLR4 exhibits differential and cell-specific responses to a variety of stimulatory factors. The complex cellular makeup of adipose tissue raises an intriguing question regarding the role of TLR4 in promoting obesity-induced inflammation and adipose tissue dysfunction. Are the metabolic effects of TLR4 due to its activation in classical insulin-sensitive tissues or in immune cells? Or simultaneously in both?

Several experimental models, including the use of bone marrow transplants (BMTs) to generate TLR4 chimeras where TLR4 is either present or absent from the whole body or from the BM-derived cells, have been developed to explore the cell-specific effects of TLR4 in obesity [21,29,31], but these studies have generated conflicting results. Saberi *et al* found that an absence of functional TLR4 in BM-derived cells conferred protection against HFD-induced insulin resistance [31]. On the other hand, Orr *et al* and Griffin *et al* found that, while TLR4 on BM-derived cells controlled the inflammatory phenotype of macrophages infiltrating the adipose tissue, it was not sufficient to modulate insulin sensitivity in the body, and non-BM-derived TLR4 played a larger role in regulating fat and liver mass [21,29]. Given such discrepancies, further investigations are required to understand the cell-specific roles of TLR4 in adipose tissue.

Due to their close structural similarity to the LPS binding moiety, it is hypothesized that FFAs can bind and stimulate TLR4. Thus, elevated plasma FFAs in obesity have the potential to activate TLR4 [14]. Shi *et al* found that *Tlr4^−/-^* mice are protected from the ability of elevated plasma FFAs to suppress insulin levels and reduce changes in glucose metabolism mediated by insulin [35]. Along with their role in promoting insulin resistance, FFAs specifically, saturated fatty acids (SFAs), activated inflammatory transcription factors NF-κB and cyclooxygenase-2 (COX-2) expression in 293 T cells and RAW264.7 cells [36]. The stimulatory effects of palmitic and stearic acid on MCP-1 secretion were observed in 3T3L1 adipocytes [37]. Consistent with these findings, previous studies in our laboratory have also shown that TLR4 is necessary for the generation of activated bone marrow derived macrophages in response to SFA [21], suggesting that the interaction between FFAs and TLR4 may play a role in macrophage polarization. In contrast to these results, other studies refute the idea that FFAs act as a direct binder and stimulator of TLR4. Studies by Lancaster *et al* suggest that palmitate, a SFA, is not a direct agonist of TLR4, instead proposing that palmitate-induced inflammation is dependent on TLR4 through its ability to alter cellular metabolism, gene expression, lipid metabolic pathways, and membrane lipid composition [38]. Despite the structural similarity of FFAs to LPS, FFA activation of TLR4 has actually been shown to take many hours in contrast to LPS activation of TLR4, which happens on the time-scale of minutes [38–41].

Palmitate-induced metabolic reprogramming in innate immune cells modulates inflammatory responses and contributes to disease progression [42]. It is critical to investigate the cell type-specific responses of TLR4 to ligands such as palmitate to better understand TLR4’s role in SFA-mediated inflammation during obesity. In this study, we investigated the adipocyte-specific effects of TLR4 through a primary culture model using ear mesenchymal stem cells (EMSCs) that are multipotent and give rise to osteocytes, chondrocytes, and adipocytes. To determine the potential role of TLR4 in inhibiting proper adipogenesis during obesity, we stimulated adipocytes derived from WT and *Tlr4^−/-^* male mice EMSCs with LPS or palmitate during their differentiation *in vitro* and assessed adipogenic and inflammatory markers. To determine the potential effects of TLR4 in the crosstalk between adipocytes and immune cells, conditioned media from LPS or palmitate treated EMSC-derived adipocytes (EMSC Ad) was further used to stimulate bone marrow dendritic cells (BMDCs) derived from WT and *Tlr4^−/-^* male mice in culture. Our *in vitro* studies with WT and *Tlr4^−/-^* BMDCs and EMSC Ad suggest a role for TLR4 activation on both macrophages and adipocytes.

## Materials and methods

### Animal models

C57Bl/6 J (WT) and *Tlr4^−/-^* (B6.B10ScN-Tlr4lps-del/JthJ; 007227) male mice on C57Bl/6 J background were purchased from Jackson Laboratories at 5 weeks of age. Animals were housed in a specific pathogen-free facility with a 12-h light/12-h dark cycle and given free access to food and water. All mice were fed *ad libitum* a diet consisting of 4.5% fat (5001; LabDiet). Animal protocols were in compliance with the Institute of Laboratory Animal Research Guide for the Care and Use of Laboratory Animals and approved by the University Committee on Use and Care of Animals at the University of Michigan (animal welfare assurance number A3114-01).

### EMSC isolation, differentiation and treatments

The primary cultures of rodent EMSC were prepared from ears of 7–8 week old wild-type and *Tlr4^−/-^* male mice [43,44]. Excised ears were washed with HBSS (Invitrogen #14,025-092) plus Primocin antibiotic (Invivogen #ant-pm-1). Minced ears were subjected to collagenase (2 mg/ml Collagenase I; Worthington Biochemical Corporation #LS004196) digestion for ½ h at 37°C. Following filtration, cells were collected via centrifugation (500 g; 7 mins at 4°C) and thereafter incubated with Red Blood Cell (RBC) Lysing Buffer for 5 mins (Sigma #R7757). Following RBC lysis, cells were resuspended in EMSC *maintenance medium*: Dulbecco’s Modified Eagle Medium/Nutrient Mixture F-12 (DMEM/F12; Invitrogen #11,330-032) supplemented with 15% Foetal Bovine Serum (FBS; Invitrogen #10,082-147) and 1 ng/ml Fibroblast Growth Factor (FGF; Peprotech) and Primocin antibiotic. Mesenchymal stem cells were plated in *maintenance medium* at a density of 0.5 × 10^6^ cells/well.

Cultured EMSC cells were differentiated into adipocytes with EMSC *differentiation media I*: DMEM/F12 supplemented with 15% FBS, 5 μg/ml Insulin (Sigma # I-5523), 1 μM Dexamethasone (Sigma # D-1756), 0.5 μM Methyl-isobutylxanthine (Sigma # I-5879), 5 μM Troglitazone (Cayman), and Penicillin Streptomycin Glutamine (Gibco # 10,378). On the third day, they were refed with EMSC *differentiation media II*: DMEM/F12 supplemented with 15% FBS, 5 μg/ml Insulin, 5 μM Troglitazone and Penicillin Streptomycin Glutamine. Cells were cultured for an additional 3 days, and then refed with *maintenance medium* and cultured for another 3–5 days.

Treatments with 10 ng/ml LPS or 200 μM of Palmitate were performed in differentiating EMSCs in d*ifferentiation media I* and was replenished upon changing the media to the EMSC *differentiation media II*. EMSC Ad experimental groups consisted of WT EMSC Ad differentiated control, WT EMSC Ad + LPS, WT EMSC Ad + Palmitate, *Tlr4^−/-^* EMSC Ad differentiated control, *Tlr4^−/-^* EMSC Ad + LPS and *Tlr4^−/-^* EMSC Ad + Palmitate. At the end of differentiation and experimental treatment, conditioned EMSC Ad media was removed and stored at −20°C for further evaluations. Cells were then either used for Oil Red O Staining or for RNA isolation.

### Preparation of fatty acids

Palmitic acid (Sigma, #P0500) was prepared in isopropanol at a stock concentration of 50 mM and then complexed with 10% BSA (endotoxin free, fatty acid free; Sigma #A8806) in isopropanol to make up 5 mM. Fatty acid free BSA was further used as control in experiments to rule out endotoxin contamination of BSA as the source of palmitic acid’s effects as reported earlier in some cases [45,46].

### Oil Red O Staining and quantification

Oil Red O Stock Solution (ORO; 0.6%) was prepared in isopropanol. Differentiated WT and *Tlr4^−/-^* EMSC Ad were fixed in 10% Buffered Neutral Formalin (VWR, #89,370-094) for 1 h at RT. After fixation, cells were washed 2X with milliQ water and 1X with 70% ethanol. Cells were stained with ORO Working Solution (1.5 parts of ORO stock to 1-part distilled water, filtered) for 15 mins at RT. Cells were then washed 2X with 70% ethanol and 1X with water. Cells were imaged for ORO staining using brightfield imaging. To measure lipid accumulation and the extent and success of adipogenesis, ORO was quantified. Isopropanol was added to stained cells and incubated for 5 mins at RT to extract ORO. ORO absorbance was measured at 520 nm on an Epoch spectrophotometer and values were plotted.

### Primary mouse BMDCs and treatment

Bone marrow cells were isolated from 7–8 week old WT and *Tlr4^−/-^* male mice femur and tibia aseptically after euthanization and cultured as described previously [47]. Cells were plated at a density of ~ 1 × 10^6^ cells per well in 12 well plates. Cells were considered to be fully differentiated to BMDCs after 6 days of incubation in DMEM (Invitrogen) containing 10% FBS (Invitrogen), penicillin/streptomycin, and GM-CSF (10 ng/ml; PeproTech). On Day 6, the media was replaced with conditioned EMSC derived adipocyte media (diluted 1:1 with BMDC media without GM-CSF) and incubated for 24 h. For both the WT and the *Tlr4^−/-^* BMDCs, 6 experimental groups were created with conditioned EMSC Ad media. WT BMDC stimulated with WT EMSC Ad Vehicle/Control Conditioned Media (WT EMSC Ad Veh → WT DC), *Tlr4^−/-^* BMDC stimulated with *Tlr4^−/-^* EMSC Ad Vehicle/Control Conditioned Media (*Tlr4^−/-^* EMSC Ad Veh → *Tlr4^−/-^* DC), WT BMDC stimulated with *Tlr4^−/-^* EMSC Ad treated with LPS (*Tlr4^−/-^* EMSC Ad LPS → WT DC), *Tlr4^−/-^* BMDC stimulated with WT EMSC Ad treated with LPS (WT EMSC Ad LPS → *Tlr4^−/-^* DC), WT BMDC stimulated with *Tlr4^−/-^* EMSC Ad treated with Palmitate (*Tlr4^−/-^* EMSC Ad Palm → WT DC), and *Tlr4^−/-^* BMDC stimulated with WT EMSC Ad treated with Palmitate (WT EMSC Ad Palm → *Tlr4^−/-^* DC). After 24 h of experimental treatment, media was removed from the BMDCs and stored at −80°C. BMDC cells were processed for RNA isolation.

### ELISA

ELISA was performed for cytokine determination of IL6, IL1β and MCP1 levels with EMSC Ad and BMDC media after respective treatments. Testing was performed by the Cancer Centre Immunology core at the University of Michigan.

### Real-time PCR

RNA was extracted from EMSC Ad and BMDC cells using RLT Buffer plus β-mercaptoethanol (Life Technologies) and cDNA was generated using a high capacity cDNA reverse transcription kit (Applied Biosystems, Foster City, CA). SYBR Green PCR Master Mix (Applied Biosystems) and the StepOnePlus system (Applied Biosystems) were used for real-time quantitative PCR. *Gapdh* expression was used as an internal control for data normalization. Primers used in the studies are provided in supplementary table 1.

### Statistical analysis

All values are reported as mean ± SEM unless otherwise stated. Statistical analyses were performed in Prism (GraphPad). One-way ANOVA was performed using Brown-Forsythe test and post hoc analysis with the unpaired two-tailed Student’s t-test. p < 0.05 was considered as statistically significant.

## Results

### *WT and* Tlr4^−/-^ *EMSC differentiation into adipocytes*

**Figure 1.**
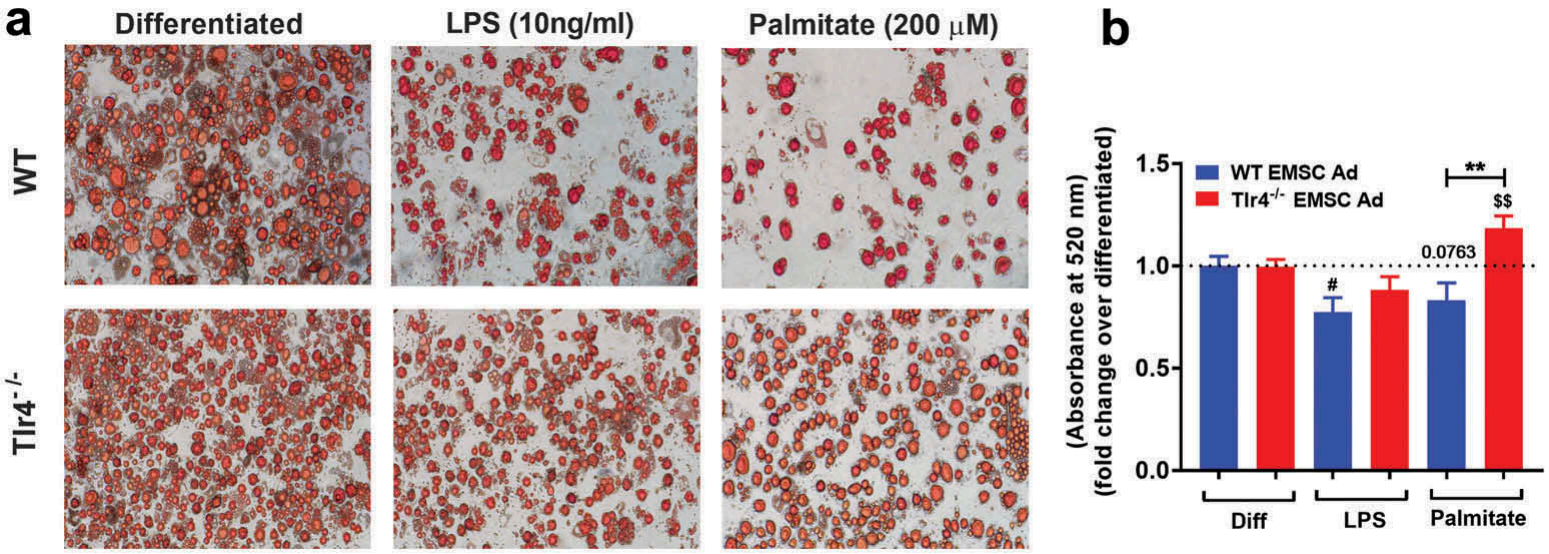
Adipogenesis in EMSC derived adipocytes. (a) Bright field images of WT EMSC derived adipocytes – TOP ROW, left- Differentiated; middle- LPS treatment (10 ng/ml); right- Palmitate treatment (200 μM). Bright field images of *Tlr4^−/-^* EMSC derived adipocytes- BOTTOM ROW, left- Differentiated; middle- LPS treatment (10 ng/ml); right- Palmitate treatment (200 μM). (b) Graph depicting ORO absorbance in WT (blue bar) and *Tlr4^−/-^* (red bar) EMSC Ad treated with LPS and palmitate. N = 6 per condition; Experiments were performed in duplicates and data is representative of three independent experiments. One-way ANOVA and Student’s t-test was performed for (B). Statistical significance is indicated by *p < 0.05, **p < 0.01, ***p < 0.001, ****p < 0.0001; error bars are SEM. Comparisons of WT Diff vs WT LPS are shown as ^#^p < 0.05, WT diff vs WT Palmitate as p = 0.0763, *Tlr4^−/-^*Diff vs *Tlr4^−/-^* Palmitate are shown as ^$$^p < 0.01. Diff = Differentiated; Ad = adipocyte

EMCSs derived from the outer ear of adult mice are multipotent stem cells and have the capacity to differentiate into osteocytes, chondrocytes, and adipocytes. The facile differentiation of EMSCs into adipocytes has previously been demonstrated [43,48], making them a good model system for our studies. The phenotype of the differentiated EMSCs with the adipogenic medium resembled the mature adipocyte with the presence of lipid accumulation, that is lacking in osteocytes and chondrocytes as confirmed in previous studies [43,44]. We characterized any intrinsic differences between the differentiation of WT and *Tlr4^−/-^* EMSCs into adipocytes. Brightfield microscope images showed no obvious difference between differentiated WT and *Tlr4^−/-^* EMSC Ad (Figure 1(a), left panels). Cells began to adapt a more rounded morphology from their original spindle-like shape, and the formation of initial lipid droplets could be observed. ORO staining was used to evaluate adipogenesis in differentiated cells. Imaging showed expanded and rounded adipocyte cells with abundant numbers of lipid droplets (Figure 1(a), left panels). No obvious differences in lipid content or cellular morphology could be distinguished between differentiated WT and *Tlr4^−/-^* EMSC Ad (Figure 1(a) left panel and 1B).

### *LPS and saturated fatty acids fail to inhibit EMSC adipogenesis in* Tlr4^−/-^ *cells*

Although some prior studies have suggested a role for TLR4 in promoting FFA-induced inflammation during obesity, many of the findings in recent literature are contradictory [29,31]. Previous investigations [36,49,50] and past studies in our lab demonstrate the ability of SFAs to drive myelopoiesis in a TLR4-dependent manner [21]. Non-haematopoietic TLR4 has been implicated in mediating glucose and insulin intolerance [21,29], and since adipose tissue dysfunction is known to be involved in metabolic syndrome [2,10,13,51], we sought to assess if treatment of EMSCs during differentiation to adipocytes with LPS or palmitate would disrupt normal adipogenesis. Consistent with previous literature and our hypothesis, the adipogenesis of WT EMSC Ad stimulated with LPS appeared to be inhibited as shown by the reduction of adipocytes and lipid droplets in comparison to untreated control. LPS significantly inhibited adipocyte differentiation, as demonstrated by the changes in morphological differentiation (Figure 1(a), middle and left panels and 1B). However, the extent of inhibition by LPS was not significantly evident in the *Tlr4^−/-^* EMSC Ad as compared to control and WT and when assessed for relative lipid content by ORO (Figure 1(a,b)). Palmitate stimulation resulted in decreased relative lipid content by ORO absorbance and adipogenesis in WT EMSC Ad compared to *Tlr4^−/-^* EMSC Ad (Figure 1(a,b)). In contrast, palmitate stimulation increased lipid content in *Tlr4^−/-^* EMSC Ad (**p < 0.01).

**Figure 2.**
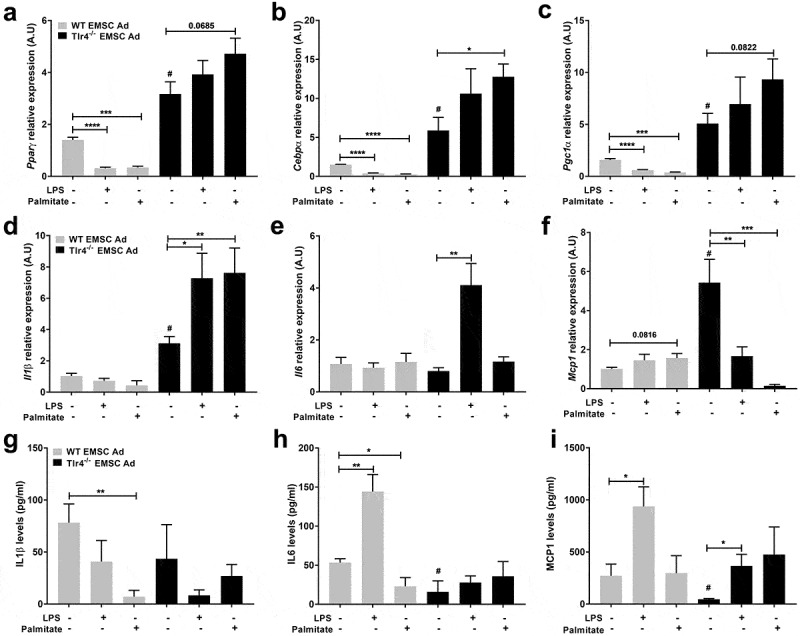
Assessment of adipogenic and inflammatory markers in WT and *Tlr4^−/-^* EMSC derived adipocytes. Relative gene expression of adipogenic markers – (a) *Pparγ* (b) *Cebpα* (c) *Pgc1α*. Relative gene expression of inflammatory markers – (d) *Il1β* (e) *Il6* (f) *Mcp1*. ELISA estimation of (g) IL1β (h) IL6 (i) MCP1. Graph depicts WT (grey bar) and *Tlr4^−/-^* (black bar) EMSC Ad treated with LPS and palmitate. N = 5–6 per condition; Experiments were performed in duplicates and data is representative of three independent experiments. One-way ANOVA and Student’s t-test was performed for (a-i). Statistical significance is indicated by *p < 0.05, **p < 0.01, ***p < 0.001, ****p < 0.0001; error bars are SEM. Comparisons of WT Differentiated control vs *Tlr4^−/-^* Differentiated control are shown as ^#^p < 0.05. Ad = adipocyte

Adipogenic markers were next assessed to investigate the effects of LPS and palmitate on WT and *Tlr4^−/-^* EMSCs differentiation. Adipogenic markers – *Pparγ, Cebpα*, and *Pgc1α* were upregulated significantly in *Tlr4^−/-^* differentiated adipocytes compared to WT (Figure 2(a–c)) suggesting a possible role for *Tlr4* in limiting adipogenesis in WT EMSC Ad. As observed earlier with ORO (Figure 1(a,b)), inhibition of adipogenesis by LPS and palmitate treatment led to decreased expression of *Pparγ, Cebpα*, and *Pgc1α* expression in WT EMSC Ad compared to WT differentiated control (Figure 2(a–c)). *Pparγ, Cebpα*, and *Pgc1α* expression were not significantly affected in *Tlr4^−/-^* EMSCs treated with LPS (Figure 2(a–c)). However, palmitate treatment led to a significant upregulation in the expression of the adipogenic marker *Cebp*α (Figure 2(b)) as observed by increased ORO absorbance in *Tlr4^−/-^* EMSC Ad (Figure 1(b)).

These results indicate that in WT adipocytes derived from EMSCs, both LPS and palmitate disrupt functional adipogenesis. In the absence of TLR4 however, LPS and palmitate fail to inhibit adipogenesis, suggesting a TLR4 dependent role in the impairment of adipogenesis.

### *Tlr4 deficiency failed to inhibit inflammatory gene expression in* Tlr4^−/-^ *EMSC Ad*

We next investigated proinflammatory gene expression as a possible cause for adipogenesis inhibition in WT EMSC Ad. *Il1β, Il6* and *Mcp1* gene expressions were evaluated in differentiated EMSCs. Interestingly, *Il1β, Il6* and *Mcp1* genes were not significantly affected by LPS or palmitate in WT EMSC Ad (Figure 2(d–f)). Contrastingly, in *Tlr4^−/-^* EMSC Ad treated with LPS, *Il1β* and *Il6* were upregulated, while *Mcp1* gene expression decreased. However, in *Tlr4^−/-^* EMSC Ad, with palmitate treatment, *Mcp1* expression was significantly downregulated, while *Il1β* was upregulated (Figure 2(d–f)). Palmitate treatment did not show any significant changes in *Il6* expression in *Tlr4^−/-^* EMSCs (Figure 2(e)). ELISA was also performed for inflammatory markers in media obtained from these treatment conditions given that the timings of the treatments may differentially influence protein and gene expression (Figure 2(g–i)). While IL6 and MCP1 cytokine levels were lower in media from untreated WT EMSC Ad, LPS treatment led to a significant increase (Figure 2(h,i)). On the other hand, in *Tlr4^−/-^* EMSC Ad, IL6 and IL1β levels were not significantly affected even with LPS and palmitate treatments (Figure 2(g,h)). MCP1 levels increased with LPS treatment but still remained lower than WT EMSC Ad (Figure 2(i)).

These results suggest that adipocytes may not be the most responsive to TLR4 binding ligands compared to macrophages. Also, there might exist a TLR4 independent mechanism to upregulate inflammatory genes in *Tlr4^−/-^* adipocytes.

**Figure 3.**
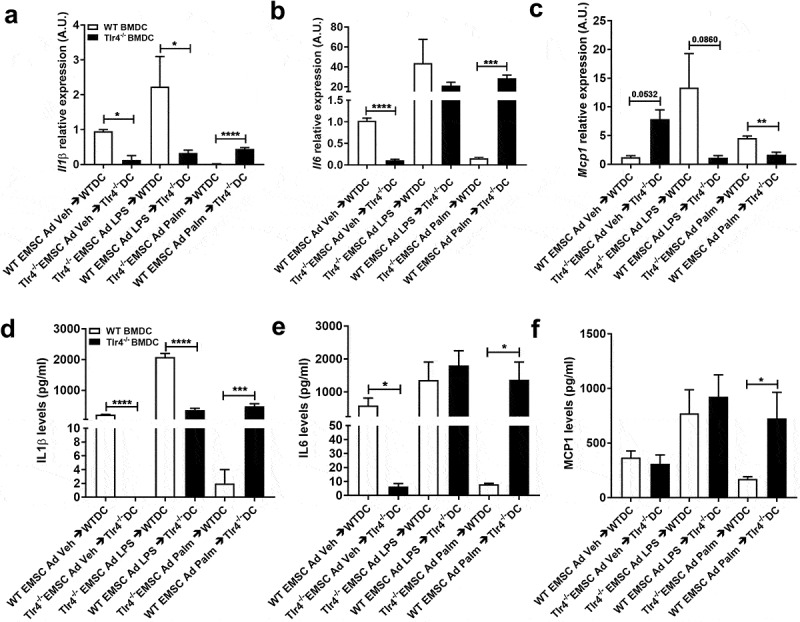
Assessment of inflammatory markers in WT and *Tlr4^−/-^* BMDCs treated with EMSC derived adipocyte conditioned media. Relative gene expression of inflammatory markers – (a) *Il1β* (b) *Il6* (c) *Mcp1*. ELISA estimation of (d) IL1β (e) IL6 (f) MCP1 in BMDC (DC) after treatment with EMSC derived adipocyte (ad) conditioned media. The experimental groups are: WT BMDC stimulated with WT EMSC Ad Vehicle/Control Conditioned Media (WT EMSC Ad Veh → WTDC), *Tlr4^−/-^* BMDC stimulated with *Tlr4^−/-^* EMSC Ad Vehicle/Control Conditioned Media (*Tlr4^−/-^* EMSC Ad Veh → *Tlr4^−/-^* DC), WT BMDC stimulated with *Tlr4^−/-^* EMSC Ad treated with LPS (*Tlr4^−/-^* EMSC Ad LPS → WTDC), *Tlr4^−/-^* BMDC stimulated with WT EMSC Ad treated with LPS (WT EMSC Ad LPS → *Tlr4^−/-^* DC), WT BMDC stimulated with *Tlr4^−/-^* EMSC Ad treated with Palmitate (*Tlr4^−/-^* EMSC Ad Palm → WTDC), and *Tlr4^−/-^* BMDC stimulated with WT EMSC Ad treated with Palmitate (WT EMSC Ad Palm → *Tlr4^−/-^* DC). N = 5–6 per condition; Experiments were performed in duplicates and data is representative of 3–4 independent experiments. One-way ANOVA and Student’s t-test was performed for (a-f). Statistical significance is indicated by *p < 0.05, **p < 0.01, ***p < 0.001, ****p < 0.0001; error bars are SEM. Ad = adipocyte; DC = dendritic cells, Veh = Vehicle, Palm = Palmitate

TLR4 activation is not required in BMDCs to respond to adipocyte factors after palmitate exposure Treating cultured bone-marrow-derived macrophages or dendritic cells with SFAs recapitulates many features of macrophage polarization that are observed in the ATMs of mice consuming diets high in saturated fat, which implies a role for adipocyte-macrophage crosstalk [52,53]. We sought to investigate the potential role of TLR4 in this inflammatory crosstalk and hypothesized that conditioned culture media from LPS or palmitate treated WT or *Tlr4^−/-^* EMSC Ad might affect the *in vitro* polarization of BMDCs from WT or *Tlr4^−/-^* bone marrow cells. We therefore investigated the gene expression of inflammatory markers to assess this inflammatory response. As controls, WT EMSC Ad media was placed on WT BMDCs, and *Tlr4^−/-^* EMSC Ad media was placed on *Tlr4^−/-^* BMDCs (WT EMSC Ad→WTDC, *Tlr4^−/-^* EMSC Ad→*Tlr4^−/-^* DC), (Figure 3).

LPS conditioned media from WT adipocytes failed to upregulate *Il1β* and *Mcp1* gene expression in *Tlr4^−/-^* BMDCs (WT EMSC Ad LPS → *Tlr4^−/-^* DC) compared to LPS conditioned media from *Tlr4^−/-^* adipocytes added to WT BMDCs (*Tlr4^−/-^* EMSC Ad LPS → WT DC) (Figure 3(a–c)). The same treatment did not affect *Il6* expression significantly (Figure 3(b)). This suggests a vital requirement for TLR4 activation in BMDCs more than in adipocytes to promote an inflammatory response. These results were also confirmed from cytokine levels in the media (Figure 3(d–f)).

Opposite effects were observed in *Tlr4^−/-^* BMDCs upon exposure to palmitate conditioned media from WT adipocytes. *Il1β* and *Il6* gene expression were upregulated, while *Mcp1* expression was downregulated in *Tlr4^−/-^* BMDCs compared to palmitate conditioned media from *Tlr4^−/-^* adipocytes exposed to WT BMDCs (Figure 3(a–c)). Protein levels of these cytokines showed similar results (Figure 3(d–f)). These results suggest a TLR4 independent mechanism in BMDCs to activate the inflammatory response in the case of SFAs. It is quite possible that *Tlr4^−/-^* BMDCs were able to respond to metabolic products and adipokines produced by WT adipocytes exposed to palmitate and induce proinflammatory cytokines.

## Discussion

The synergistic interaction between adipocytes and macrophages is critical in maintaining obesity-induced adipose tissue inflammation and secondary insulin resistance. However, adipocytes and immune cells may exhibit cell-specific contributions. The aim of this study was to delineate cell type intrinsic differences in adipogenic, or inflammatory responses associated with TLR4. Our results show that the signals arising from the adipocyte compartment can influence the inflammatory status of BMDCs. Media from adipocytes exposed to LPS or palmitate promoted a distinct inflammatory response in the BMDCs.

The mechanism through which proinflammatory factors affect adipogenesis is speculated to be largely through the direct activation of NF-κB pathway, which in turn reduces PPARγ activity during the adipocyte differentiation process [54,55]. The inhibitory effects of inflammatory inducers on the adipocyte differentiation process have been studied extensively [54–56]. The earliest [57] and more recent evidence linking TLR4 to lipid-induced inflammation is through LPS [31,58,59]. In obesity and metabolic syndrome, adipose tissue often displays impaired adipogenesis, which contributes to inflammation and metabolic dysfunction [11–13]. In our approach, we mimicked an obese or inflammatory environment by performing TLR4 stimulation with LPS or palmitate in WT EMSC Ad throughout the differentiation process, and this attenuated adipogenesis as evidenced by the reduced expression of adipogenic genes. However, although WT EMSC Ad treated with LPS showed decreased lipid content by ORO absorbance compared to WT Control EMSCs, there were no significant differences between LPS treated WT and *Tlr4^−/-^* EMSC Ad in lipid content. LPS is an endotoxin, that may not necessarily increase lipid content in adipocytes but serves as a ligand for TLR4 induced inflammatory pathways [32] that may eventually inhibit adipocyte differentiation. It could be speculated that only adipocyte differentiation was affected directly by TLR4 activation or through lipid metabolism which may be in contrast to some other studies due to differences in LPS treatment periods [60]. However, the lack of cell count after the differentiation process might be a limitation in our study.

A major source of chronic inflammation in obesity is adipocyte dysfunction, which is associated with the interaction between adipocyte hypertrophy and macrophage infiltration of M1 polarized ATMs. In addition to the paracrine loop between adipocytes and macrophages, the intracellular metabolism of FFAs has also been found to control ATM function [61]. While experimental evidence supports an important role for TLR4 in adipose inflammation [31,59], this is thought to directly involve TLR4 by SFAs for macrophage polarization and differentiation but several lines of evidence also argue against SFAs being direct TLR4 agonists [38]. A previous study showed that 3T3L1 cells treated with 100 μM palmitate led to lipid accumulation but did not inhibit differentiation [62]. In our study, palmitate stimulation with 200 μM decreased relative lipid content and inhibited adipogenesis in WT EMSC Ad but failed to induce impairment in *Tlr4^−/-^* EMSC Ad suggesting a suppression of lipid homoeostasis in mature adipocytes. In *Tlr4^−/-^* EMSC Ad, palmitate may be more readily incorporated into differentiating adipocytes, leading to heightened lipid content and enhanced adipogenic gene expression. However, in WT EMSC Ad, excess palmitate incorporation might not directly trigger inflammatory cytokine levels but might lead to increased apoptosis [63] and de novo synthesis of ceramides [64] thereby hampering efficient adipocyte differentiation. This suggests an indirect TLR4-dependent and independent role for palmitate in adipocytes. These findings are consistent with previous studies that have demonstrated a TLR4-dependent effect of FFA-induced inflammation [21,49,65], and in contrast to those that have suggested that TLR4 does not play a direct role in FFA-induced inflammation [38].

TLR2 has also been shown to recognize and respond to LPS, indicating that TLR2 and TLR4 may work synergistically to respond to inflammatory signals from the environment [33,65]. Further studies are required to address this question in adipocytes. A limitation of our study is that while we used fatty acid free BSA in order to rule out endotoxin contamination of BSA, this may still be an issue effecting results [45,46]. Additionally, other control groups might be required to further study the complexity of TLR4 signalling including shorter treatment times with LPS and palmitate, treatment pre- and post differentiation, treatment with unsaturated fatty acids and further combinations of WT and *Tlr4^−/-^* BMDCs and adipocytes.

Studies until now have relied on inflammatory changes in macrophages to modulate adipocyte function. However, adipocyte related dysfunction itself potentially influences macrophage phenotypes. With our evaluations of continuously treating adipocytes with TLR4 ligands during adipocyte differentiation, we tried to mimic the maintenance of an obesogenic environment. We hypothesized that the adipocyte micro‐environment, might be important regulators in shaping macrophage functions and phenotype. Palmitate induces lipotoxicity with the overproduction of reactive oxygen species (ROS) and when converted to palmitoyl-CoA also serves as a precursor for ceramides generation, which further enhance ROS production [42]. Previous work from our lab showed that palmitic acid stimulated WT lineage negative bone marrow cells but not *Tlr4^−/-^* bone marrow cells when directly added to the cells [21]. However, in the current study, we observed that conditioned media from palmitate treated WT adipocytes was able to stimulate inflammatory changes in *Tlr4^−/-^* BMDCs suggesting the contribution of adipocyte palmitate derived metabolites in the modulation of macrophage phenotype. A limitation is that although the conditioned media from the adipocytes was diluted prior to treatment of BMDCs, there might still be the presence of residual LPS or palmitate in our experiments. However, our data suggests that adipocyte associated cytokines and metabolites could alter the TLR4 response in a cell-specific manner either through a TLR4-dependent or independent pathway. In obesity, the level of circulating SFAs, particularly palmitate is elevated and is responsible for promoting tissue dysfunction and our studies suggest that the crosstalk between macrophages and surrounding cells are critical for this dysfunction.

Inflammatory cytokines may promote dysregulation of fatty acid metabolism in WAT. IL6 might interfere with adipocyte function by enhancing mesenchymal stem cell proliferation, maintaining the cells in an undifferentiated state and inhibiting adipogenesis [66]. Additionally, IL6 can directly affect lipid metabolism and activate pathways to promote increased energy turnover. In humans, IL6 stimulates lipolysis, increases FFA concentrations and upregulates whole-body fat oxidation [57,67]. It is known that in obesity, adipose tissue represents a major source of increased circulating IL6 [68], but the relevant adipose-tissue cell type is unclear because *Il6* mRNA is expressed by adipocytes, ATMs, and other adipose-tissue cell types [69]. Our observations highlight the complexity in determining cell intrinsic responses since the sources of inflammatory cytokines such as IL6 and IL1β are unclear. We observed varied expression of IL6 protein and mRNA that might be attributed to longer treatment times for LPS and palmitate. Therefore, a limitation of our study was the lack of assessment of cytokines at shorter time frames during the differentiation process making it challenging to interpret the protein and gene expression levels. A recent study showed that adipocyte IL6 potently promotes ATM accumulation in the absence of major changes in glucose or insulin tolerance. In contrast, myeloid cell IL6 suppressed M1 macrophage polarization, reduced ATM accumulation, and improved tolerance to both glucose and insulin [70]. These studies and ours suggest the importance of assessing both cytokine protein and mRNA levels to understand the inflammatory response.

Our data demonstrates that adipose tissue inflammation is far more complex than simply a function of ATM accumulation. Overall, our studies demonstrate that TLR4 plays an important role in LPS and SFA-mediated adipocyte dysfunction through impairment of adipogenesis. Our studies show that adipocyte TLR4 might not elicit a potent inflammatory response to LPS or palmitate, but adipocyte metabolism associated mediators might be involved in the adipocyte-immune cell inflammatory cross-talk in a TLR4-dependent and -independent manner. Excess circulating palmitate is a potential dietary trigger resulting in the induction of chronic inflammation, such as that seen in type 2 diabetes. Further investigations are required to decipher the pathological role of palmitate in inflammatory associated diseases as palmitate uptake may control cellular lipid homoeostasis in physiological conditions. Thus, given the intricacy of adipose tissue inflammatory responses, this study emphasizes the need to delineate and clarify the role of various cell types and their interactions in obesity and associated metabolic diseases to strategize new potential therapeutic approaches in the future.

## Supplementary Material

Supplemental Material
