# Supplementary material for: Role of TLR4 in the induction of inflammatory changes in adipocytes and macrophages
Source: Adipocyte. 2020 May 13;9(1):212–22. doi: 10.1080/21623945.2020.1760674 (PMC7238871; doi:10.1080/21623945.2020.1760674)
Supplement: Supplemental Material [file KADI_A_1760674_SM6964.docx]

|  | **Forward Primer** | **Reverse Primer** |
| --- | --- | --- |
| *Gapdh* | TGAAGCAGGCATCTGAGGG | CGAAGGTGGAAGAGTGGGAG |
| *Il6* | TAGTCCTTCCTACCCCAATTTCC | AAGGAACCCTTAGAGTGCTTACT |
| *Mcp1* | TTAAAAACCTGGATCGGAACCAA | GCATTAGCTTCAGATTTACGGGT |
| *Pgc1α* | TATGGAGTGACATAGAGTGTGCT | CCACTTCAATCCACCCAGAAAG |
| *Pparγ* | GGAAGACCACTCGCATTCCTT | TCGCACTTTGGTATTCTTGGAG |
| *Cebpα* | CAAGAACAGCAACGAGTACCG | GTCACTGGTCAACTCCAGCAC |

**Supplementary Table 1. Primer sequences for RT-PCR**
